# 2,2′-(Imino­dimethyl­ene)dibenz­imid­azol­ium bis­(perchlorate) methanol solvate

**DOI:** 10.1107/S1600536808008519

**Published:** 2008-04-02

**Authors:** Chun-Shan Zhou, Xue-Ying Huang, Xiang-Gao Meng

**Affiliations:** a Key Laboratory of Pesticides and Chemical Biology, Ministry of Education, College of Chemistry, Central China Normal University, Wuhan 430079, People’s Republic of China

## Abstract

In the title compound, C_16_H_17_N_5_^2+^·2ClO_4_^−^·CH_3_OH, the dihedral angle between the two benzimidazolium ring systems is 34.6 (1)°. The anions and solvent mol­ecules are linked to the cation by N—H⋯O hydrogen bonds. In the crystal structure, the combination of N—H⋯O and O—H⋯O hydrogen bonds results in two-dimensional layers running parallel to the (010) plane; these are in turn linked by π–π inter­actions, forming a three-dimensional network.

## Related literature

For related literature, see: Adams *et al.* (1990 ▶); Allen (2002 ▶); Berends & Stephan (1984 ▶); Bernstein *et al.* (1995 ▶); Bruno *et al.* (2002 ▶); Girasolo *et al.* (2000 ▶); Liao *et al.* (2001 ▶); Liu *et al.* (2004 ▶); Meng *et al.* (2005 ▶, 2006*a* ▶,*b* ▶); Spek (2003 ▶); Tarazon Navarro & McKee (2003 ▶); Xu *et al.* (2007 ▶); Young *et al.* (1995 ▶); Zheng *et al.* (2005 ▶).
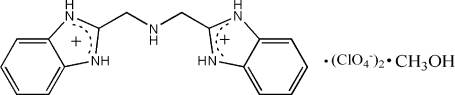


## Experimental

### 

#### Crystal data

C_16_H_17_N_5_^2+^·2ClO_4_^−^·CH_4_O
*M* *_r_* = 510.29
Monoclinic, 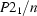

*a* = 8.3359 (4) Å
*b* = 18.0323 (8) Å
*c* = 14.8532 (7) Å
β = 102.944 (1)°
*V* = 2175.89 (18) Å^3^
*Z* = 4
Mo *K*α radiation
μ = 0.36 mm^−1^
*T* = 295 (2) K
0.30 × 0.20 × 0.20 mm

#### Data collection

Bruker SMART APEX CCD area-detector diffractometer
Absorption correction: multi-scan (*SADABS*; Sheldrick, 1997 ▶) *T*_min_ = 0.900, *T*_max_ = 0.932
24553 measured reflections
4957 independent reflections
3479 reflections with *I* > 2σ(*I*)
*R*_int_ = 0.069

#### Refinement

*R*[*F* ^2^ > 2σ(*F* ^2^)] = 0.057
*wR*(*F* ^2^) = 0.169
*S* = 1.02
4957 reflections
317 parameters
5 restraints
H atoms treated by a mixture of independent and constrained refinement
Δρ_max_ = 0.26 e Å^−3^
Δρ_min_ = −0.23 e Å^−3^

### 

Data collection: *SMART* (Bruker, 2001 ▶); cell refinement: *SAINT-Plus* (Bruker, 2001 ▶); data reduction: *SAINT-Plus*; program(s) used to solve structure: *SHELXS97* (Sheldrick, 2008 ▶); program(s) used to refine structure: *SHELXL97* (Sheldrick, 2008 ▶); molecular graphics: *PLATON* (Spek, 2003 ▶); software used to prepare material for publication: *PLATON*.

## Supplementary Material

Crystal structure: contains datablocks global, I. DOI: 10.1107/S1600536808008519/wn2246sup1.cif

Structure factors: contains datablocks I. DOI: 10.1107/S1600536808008519/wn2246Isup2.hkl

Additional supplementary materials:  crystallographic information; 3D view; checkCIF report

## Figures and Tables

**Table 1:** Hydrogen-bond geometry (Å, °)

*D*—H⋯*A*	*D*—H	H⋯*A*	*D*⋯*A*	*D*—H⋯*A*
N1—H1⋯O7^i^	0.86 (3)	2.42 (3)	3.200 (3)	150 (2)
N2—H2*A*⋯O8	0.831 (16)	2.15 (2)	2.895 (3)	150 (3)
N2—H2*A*⋯O9	0.831 (16)	2.489 (19)	3.233 (3)	150 (2)
N3—H3*A*⋯O1	0.831 (16)	1.996 (17)	2.799 (3)	162 (2)
N4—H4*A*⋯O1	0.857 (16)	1.985 (17)	2.823 (3)	166 (2)
N5—H5*A*⋯O4	0.824 (16)	2.46 (2)	3.197 (4)	150 (2)
N5—H5*A*⋯O5	0.824 (16)	2.21 (2)	2.939 (3)	147 (2)
O1—H1*C*⋯O6^ii^	0.805 (17)	2.00 (2)	2.765 (3)	159 (3)

Symmetry codes: (i) 
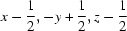
; (ii) 
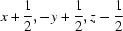
.

**Table 2:** Table 2 π–π Stacking interactions (°, Å)

*Cg*_*i*_	*Cg*_*j*_	Dihedral angle	CCD	Interplanar spacing
*Cg*1	*Cg*2^iv^	0.42	3.854 (2)	3.349 (2)
*Cg*1	*Cg*4^iv^	0.35	3.557 (2)	3.354 (2)
*Cg*3	*Cg*2^iv^	0.85	3.612 (2)	3.360 (2)
*Cg*3	*Cg*4^v^	0.36	3.929 (2)	3.453 (2)

CCD is the centroid-to-centroid distance; *Cg*1 is the centroid of atoms N2/N3/C2/C3/C8; *Cg*2 is the centroid of atoms N4/N5/C10/C11/C16; *Cg*3 is the centroid of atoms C3–C8; *Cg*4 is the centroid of atoms C11–C16. Symmetry codes: (iv) 
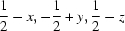
; (v) 
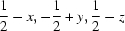
.

**Fig. 1.**
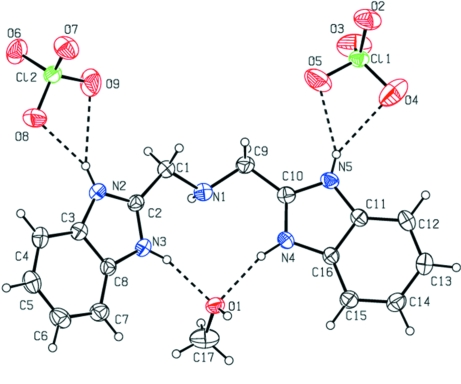
Molecular structure of the title compound, showing the atom-numbering scheme. Displacement ellipsoids are drawn at the 30% probability level and H atoms as spheres of arbitrary radius. Hydrogen bonds are shown as dashed lines.

**Fig. 2.**
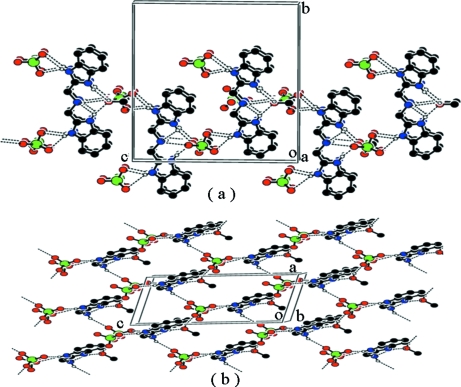
Part of the crystal structure of the title compound, showing the formation of the two-dimensional network parallel to the (010) plane, built from N—H···O and O—H···O hydrogen bonds which are shown as dashed lines. (a) the view along the a axis and (b) the view along the b axis. For the sake of clarity, H atoms not involved in hydrogen bonding have been omitted.

## supplementary crystallographic information

### Comment

Bis[N-(benzimidazol-2-ylmethyl)]amine (IDB) and its analogs have been utilized extensively in the synthesis of various metal complexes to mimic certain biological activities; these include superoxide dismutase (Liao *et al.*, 2001), DNA probe (Girasolo *et al.*, 2000), alkaline phosphatase (Young *et al.*, 1995). In IDB, each benzimidazole (bzim) arm possesses one imine N atom, one amine NH group and, at the centre, an NH group. The two imine N atoms can chelate metal ions, while the three NH groups can act as hydrogen bond donors. In the absence of metal coordination, the two imine N atoms can also act as hydrogen bond acceptors. The easily formed coordination and hydrogen-bonding interactions allow the central acyclic —CH_2_—NH—CH_2_– unit to possess various steric arrangements. In our and other reported organic complex analogs (Meng *et al.*, 2006*a*; Meng *et al.*, 2006*b*; Meng *et al.*, 2005; Zheng *et al.*, 2005; Liu *et al.*, 2004; Tarazon Navarro *et al.*, 2003) we find that IDB preferentially adopts a more extended conformation, with the two bzim groups pointing away from each other. However, in most examples of metal-complexes it adopts a more crowded conformation, with the two bzim units bending towards the same side of the central acyclic linkage (Berends & Stephan, 1984; Xu *et al.*,  2007; Adams *et al.*,  1990). With the aim of gaining more insight into the influence of solvents and anions on the crystal structure, we have synthesized H_2_IDB^2+^.2ClO_4_^-^.CH_3_OH and report its molecular and supramolecular structure in this communication.

The two imine N atoms on both bzim arms are protonated, as confirmed by the residual electron peaks around the imine N atoms during the structure refinement. The positive charge on each imine N atom is delocalized in the imidazole ring, as evidenced by the near equivalence of bonds C2—N2/C2—N3 [1.323 (3)/1.320 (3) Å] and C10—N4/C10—N5 [1.330 (3)/1.32 6(3) Å]. These values lie within the range of C—N single bond [1.357 (2) Å] and C═N double bond [1.318 (2) Å] determined at low temperature by Tarazon Navarro & McKee (2003). In the title compound, the dication adopts a somewhat folded conformation and the dihedral angle between two bzim groups is 34.6 (1)°. This angle is comparable with those in (IDB).(H_2_O)_4_ [36.2 (1)° and 39.7 (1) °; Meng *et al.* (2006*a*)] and (IDB)_2_.(H_2_O)_2_.C_2_H_5_OH [26.8 (1)° and 25.8 (1)°; Meng *et al.* (2006*b*)], but considerably larger those in IDB [0.0°; Tarazon Navarro & McKee (2003)], HIDB^+^.Cl^-^ [5.4 (1)°; Zheng *et al.* (2005)], HIDB^+^.ClO_4_^-^ [3.7 (1)°; Liu *et al.* (2004)] and H_2_IDB^2+^.SO_4_^2-^ [3.3 (1)°; Meng *et al.* (2005)].

Two imine N atoms (N2 and N5) act as hydrogen bond donors, *via* atoms H2A and H5A, respectively, to atoms O8/O9 and O4/O5, thereby generating four hydrogen bonds, each two forming an *R*^2^_1_(4) (Fig.1) motif (Bernstein *et al.*, 1995). The other two N atoms (N3 and N4) on bzim also act as hydrogen bond donors, forming intermolecular hydrogen bonds of *R*^1^_2_(10) motif, to the methanol solvent molecule. The methanol molecule donates its hydroxyl H atom to atom O6, forming a relatively strong O—H···O hydrogen bond. There is a pseudo-mirror plane passing through the central NH group and the solvent C and O atoms.

In the crystal structure, the component ions are assembled into a three-dimensional network by a combination of N—H···O, O—H···O hydrogen bonds and π-π interactions which can be analyzed in terms of several substructures. Firstly, by the six cooperative hydrogen-bonding interactions (Table 1), the discrete dications, anions and methanol molecules are joined together, forming a relatively independent neutral unit. These neutral units are linked together by N1···O7 (-1/2 + *x*,1/2 - *y*,-1/2 + *z*) and O1···O6 (1/2 + *x*,1/2 - *y*,-1/2 + *z*) hydrogen bonds related by the n-glide plane at *y* =1/4, forming a two-dimensional layer parallel to the (010) plane in the domain of -0.259 < *y* < 0.759 (Fig.2). Secondly, by π–π stacking interactions, adjacent two-dimensional layers are interlinked into a simple three-dimensional network. The geometric details of the π–π stacking interactions are listed in Table 2. A CSD (Version 1.9, September 2006 release; Allen, 2002; Bruno *et al.*, 2002) study indicates that π–π stacking interactions play a critical role in stabilizing the crystal structures of organic and metal-organic compounds containing poly-bzim groups. For instance, in HIDB^+^.ClO_4_^- ^ (Liu *et al.*,  2004), the N—H···N and N—H···O hydrogen bonds link the component ions into one-dimensional chains. However, π–π stacking interactions between adjacent bzim groups link the molecules into a three-dimensional network.

### Experimental

All the reagents and solvents were used as obtained without further purification. Bis(benzimidazol-2-yl-methyl)amine (IDB) was prepared according to the method described by Adams *et al.* (1990). 2 g of the powdered title compound were dissolved in 15 ml methanol and adjusted to pH 5 using HClO_4_. Colorless crystals were obtained as blocks by slowly evaporating the solvent over a period of several days.

### Refinement

H atoms bonded to C atoms were located in difference maps and subsequently treated as riding, with C—H distances of 0.93 Å (aromatic), 0.97 Å (methylene) and 0.96 Å (methyl); *U*_iso_(H) = 1.2*U*_eq_(aromatic and methylene C) or 1.5*U*_eq_(methyl C). H atoms bonded to N and methanol O atoms were also found in difference maps, and refined with restraints of N—H = 0.86 (2) Å and O—H = 0.82 (2) Å; *U*_iso_(H) values were set equal to k times those of their carrier atoms (k=1.2 for N and 1.5 for O atoms)

### Crystal data

**Table:**

C_16_H_17_N_5_^2+^·2ClO_4_^–^·CH_4_O	*F*_000_ = 1056
*M**_r_* = 510.29	*D*_x_ = 1.558 Mg m^−^^3^
Monoclinic, *P*2_1_/*n*	Mo *K*α radiation λ = 0.71073 Å
Hall symbol: -P 2yn	Cell parameters from 7139 reflections
*a* = 8.3359 (4) Å	θ = 2.3–25.2º
*b* = 18.0323 (8) Å	µ = 0.36 mm^−^^1^
*c* = 14.8532 (7) Å	*T* = 295 (2) K
β = 102.944 (1)º	Block, colorless
*V* = 2175.89 (18) Å^3^	0.30 × 0.20 × 0.20 mm
*Z* = 4	

### Data collection

**Table:**

Bruker SMART APEX CCD area-detector diffractometer	4957 independent reflections
Radiation source: fine focus sealed Siemens Mo tube	3479 reflections with *I* > 2σ(*I*)
Monochromator: graphite	*R*_int_ = 0.069
*T* = 295(2) K	θ_max_ = 27.5º
0.3° wide ω exposures scans	θ_min_ = 1.8º
Absorption correction: multi-scan(SADABS; Sheldrick, 1997)	*h* = −10→10
*T*_min_ = 0.900, *T*_max_ = 0.932	*k* = −23→21
24553 measured reflections	*l* = −19→19

### Refinement

**Table:**

Refinement on *F*^2^	Secondary atom site location: difference Fourier map
Least-squares matrix: full	Hydrogen site location: inferred from neighbouring sites
*R*[*F*^2^ > 2σ(*F*^2^)] = 0.057	H atoms treated by a mixture of independent and constrained refinement
*wR*(*F*^2^) = 0.169	*w* = 1/[σ^2^(*F*_o_^2^) + (0.1086*P*)^2^] where *P* = (*F*_o_^2^ + 2*F*_c_^2^)/3
*S* = 1.02	(Δ/σ)_max_ = 0.001
4957 reflections	Δρ_max_ = 0.26 e Å^−^^3^
317 parameters	Δρ_min_ = −0.23 e Å^−^^3^
5 restraints	Extinction correction: none
Primary atom site location: structure-invariant direct methods	

### Special details

**Table:**

Geometry. All e.s.d.'s (except the e.s.d. in the dihedral angle between two l.s. planes) are estimated using the full covariance matrix. The cell e.s.d.'s are taken into account individually in the estimation of e.s.d.'s in distances, angles and torsion angles; correlations between e.s.d.'s in cell parameters are only used when they are defined by crystal symmetry. An approximate (isotropic) treatment of cell e.s.d.'s is used for estimating e.s.d.'s involving l.s. planes.
Refinement. Refinement of *F*^2^ against ALL reflections. The weighted *R*-factor *wR* and goodness of fit *S* are based on *F*^2^, conventional *R*-factors *R* are based on *F*, with *F* set to zero for negative *F*^2^. The threshold expression of *F*^2^ > σ(*F*^2^) is used only for calculating *R*-factors(gt) *etc*. and is not relevant to the choice of reflections for refinement. *R*-factors based on *F*^2^ are statistically about twice as large as those based on *F*, and *R*- factors based on ALL data will be even larger.

### Fractional atomic coordinates and isotropic or equivalent isotropic displacement parameters (Å^2^)

**Table:**

	*x*	*y*	*z*	*U*_iso_*/*U*_eq_	
C1	0.2809 (3)	0.30786 (14)	0.38370 (17)	0.0497 (6)	
H1A	0.1809	0.2888	0.3983	0.060*	
H1B	0.3586	0.3183	0.4414	0.060*	
C2	0.3517 (3)	0.25067 (13)	0.33135 (15)	0.0396 (5)	
C3	0.4580 (3)	0.14439 (14)	0.29713 (16)	0.0426 (5)	
C4	0.5154 (3)	0.07295 (14)	0.29609 (19)	0.0519 (6)	
H4	0.5108	0.0394	0.3430	0.062*	
C5	0.5799 (3)	0.05391 (15)	0.2218 (2)	0.0582 (7)	
H5	0.6225	0.0065	0.2190	0.070*	
C6	0.5829 (4)	0.10337 (16)	0.1514 (2)	0.0605 (7)	
H6	0.6244	0.0875	0.1015	0.073*	
C7	0.5271 (3)	0.17525 (15)	0.15176 (17)	0.0520 (6)	
H7	0.5315	0.2085	0.1045	0.062*	
C8	0.4636 (3)	0.19489 (13)	0.22791 (15)	0.0411 (5)	
C9	0.2625 (4)	0.44157 (14)	0.38863 (18)	0.0543 (7)	
H9A	0.3339	0.4307	0.4482	0.065*	
H9B	0.1560	0.4563	0.3987	0.065*	
C10	0.3339 (3)	0.50353 (14)	0.34394 (15)	0.0418 (5)	
C11	0.4340 (3)	0.61419 (13)	0.32023 (16)	0.0409 (5)	
C12	0.4887 (3)	0.68676 (14)	0.32554 (17)	0.0499 (6)	
H12	0.4805	0.7175	0.3746	0.060*	
C13	0.5561 (3)	0.71112 (15)	0.25419 (18)	0.0541 (7)	
H13	0.5960	0.7594	0.2555	0.065*	
C14	0.5658 (3)	0.66542 (15)	0.18039 (18)	0.0531 (6)	
H14	0.6096	0.6845	0.1329	0.064*	
C15	0.5131 (3)	0.59301 (14)	0.17489 (17)	0.0489 (6)	
H15	0.5213	0.5625	0.1256	0.059*	
C16	0.4468 (3)	0.56799 (13)	0.24711 (15)	0.0393 (5)	
C17	0.2881 (5)	0.3822 (2)	0.0512 (2)	0.0840 (11)	
H17A	0.1777	0.3890	0.0588	0.126*	
H17B	0.2965	0.3352	0.0223	0.126*	
H17C	0.3151	0.4212	0.0132	0.126*	
Cl1	0.24642 (8)	0.62635 (4)	0.59521 (4)	0.0481 (2)	
Cl2	0.31148 (8)	0.10820 (3)	0.57634 (4)	0.0464 (2)	
N1	0.2440 (3)	0.37571 (11)	0.33106 (14)	0.0445 (5)	
H1	0.145 (4)	0.3694 (14)	0.2981 (19)	0.053*	
N2	0.3856 (3)	0.18181 (11)	0.36014 (13)	0.0445 (5)	
H2A	0.358 (3)	0.1635 (15)	0.4057 (14)	0.053*	
N3	0.3963 (2)	0.26023 (11)	0.25234 (13)	0.0418 (5)	
H3A	0.388 (3)	0.3016 (10)	0.2272 (16)	0.050*	
N4	0.3827 (2)	0.49959 (11)	0.26472 (13)	0.0430 (5)	
H4A	0.380 (3)	0.4598 (11)	0.2328 (15)	0.052*	
N5	0.3622 (3)	0.57129 (12)	0.37846 (13)	0.0445 (5)	
H5A	0.326 (3)	0.5852 (15)	0.4229 (14)	0.053*	
O1	0.3990 (2)	0.38398 (9)	0.13891 (12)	0.0471 (4)	
H1C	0.493 (2)	0.3935 (16)	0.138 (2)	0.071*	
O2	0.3285 (3)	0.62766 (13)	0.68957 (15)	0.0858 (7)	
O3	0.0789 (3)	0.63840 (17)	0.58637 (17)	0.1044 (9)	
O4	0.3121 (3)	0.67790 (18)	0.5420 (2)	0.1118 (10)	
O5	0.2696 (4)	0.55666 (15)	0.55727 (18)	0.1208 (11)	
O6	0.1832 (3)	0.06270 (12)	0.59527 (17)	0.0829 (7)	
O7	0.4415 (3)	0.11275 (12)	0.65578 (14)	0.0788 (7)	
O8	0.3685 (3)	0.07408 (13)	0.50223 (13)	0.0738 (6)	
O9	0.2500 (3)	0.17971 (12)	0.54776 (17)	0.0911 (8)	

### Atomic displacement parameters (Å^2^)

**Table:**

	*U*^11^	*U*^22^	*U*^33^	*U*^12^	*U*^13^	*U*^23^
C1	0.0628 (17)	0.0430 (14)	0.0466 (13)	−0.0040 (12)	0.0194 (12)	0.0000 (11)
C2	0.0370 (12)	0.0417 (13)	0.0399 (11)	−0.0063 (10)	0.0083 (9)	0.0020 (10)
C3	0.0385 (13)	0.0436 (14)	0.0443 (12)	−0.0009 (10)	0.0063 (10)	0.0023 (10)
C4	0.0530 (16)	0.0385 (14)	0.0611 (15)	−0.0012 (12)	0.0063 (12)	0.0027 (12)
C5	0.0556 (17)	0.0457 (16)	0.0699 (18)	0.0077 (13)	0.0071 (14)	−0.0067 (13)
C6	0.0521 (17)	0.0680 (19)	0.0644 (17)	0.0004 (14)	0.0195 (14)	−0.0169 (15)
C7	0.0494 (15)	0.0573 (17)	0.0523 (14)	−0.0034 (12)	0.0179 (12)	−0.0014 (12)
C8	0.0393 (13)	0.0391 (13)	0.0448 (12)	−0.0058 (10)	0.0090 (10)	−0.0014 (10)
C9	0.0732 (18)	0.0445 (15)	0.0523 (14)	0.0051 (13)	0.0293 (13)	0.0020 (11)
C10	0.0393 (13)	0.0446 (14)	0.0425 (11)	0.0077 (10)	0.0111 (9)	0.0020 (10)
C11	0.0379 (13)	0.0408 (13)	0.0434 (12)	0.0064 (10)	0.0080 (10)	−0.0020 (10)
C12	0.0479 (15)	0.0427 (14)	0.0573 (15)	0.0036 (11)	0.0077 (12)	−0.0119 (11)
C13	0.0505 (16)	0.0418 (15)	0.0689 (17)	−0.0020 (12)	0.0110 (13)	0.0017 (12)
C14	0.0544 (16)	0.0513 (16)	0.0579 (15)	0.0056 (12)	0.0217 (12)	0.0084 (12)
C15	0.0526 (16)	0.0464 (15)	0.0514 (14)	0.0049 (12)	0.0199 (12)	−0.0006 (11)
C16	0.0389 (12)	0.0353 (13)	0.0444 (12)	0.0070 (9)	0.0108 (10)	−0.0002 (9)
C17	0.073 (2)	0.125 (3)	0.0535 (17)	0.0090 (19)	0.0120 (16)	0.0070 (17)
Cl1	0.0421 (4)	0.0612 (4)	0.0436 (3)	−0.0032 (3)	0.0155 (3)	−0.0038 (3)
Cl2	0.0510 (4)	0.0450 (4)	0.0454 (3)	0.0006 (3)	0.0152 (3)	0.0041 (2)
N1	0.0399 (12)	0.0494 (13)	0.0428 (11)	0.0018 (9)	0.0065 (9)	0.0017 (9)
N2	0.0504 (12)	0.0419 (12)	0.0428 (10)	−0.0047 (9)	0.0136 (9)	0.0048 (9)
N3	0.0438 (11)	0.0364 (11)	0.0450 (11)	−0.0017 (9)	0.0099 (9)	0.0074 (8)
N4	0.0492 (12)	0.0371 (11)	0.0450 (10)	0.0043 (9)	0.0157 (9)	−0.0054 (8)
N5	0.0499 (12)	0.0450 (12)	0.0419 (11)	0.0085 (9)	0.0171 (9)	−0.0037 (9)
O1	0.0502 (11)	0.0517 (11)	0.0431 (9)	−0.0006 (8)	0.0184 (8)	0.0030 (7)
O2	0.0842 (17)	0.118 (2)	0.0479 (12)	−0.0101 (13)	−0.0010 (11)	−0.0106 (11)
O3	0.0471 (14)	0.181 (3)	0.0882 (17)	0.0107 (15)	0.0230 (12)	0.0009 (16)
O4	0.0934 (19)	0.131 (2)	0.1124 (19)	−0.0257 (16)	0.0253 (16)	0.0449 (17)
O5	0.195 (3)	0.090 (2)	0.0883 (17)	0.0224 (19)	0.0562 (19)	−0.0256 (14)
O6	0.0794 (15)	0.0663 (15)	0.1205 (18)	−0.0110 (11)	0.0595 (14)	−0.0011 (12)
O7	0.0794 (16)	0.0883 (16)	0.0576 (12)	0.0054 (12)	−0.0081 (11)	−0.0067 (10)
O8	0.0842 (15)	0.0842 (15)	0.0617 (12)	−0.0034 (12)	0.0350 (11)	−0.0092 (11)
O9	0.119 (2)	0.0498 (13)	0.0938 (17)	0.0148 (13)	0.0018 (14)	0.0128 (11)

### Geometric parameters (Å, °)

**Table:**

N1—C1	1.448 (3)	C11—C16	1.392 (3)
N1—C9	1.451 (3)	C12—C13	1.378 (3)
N2—C2	1.323 (3)	C12—H12	0.9300
N2—C3	1.396 (3)	C13—C14	1.388 (4)
N3—C2	1.320 (3)	C13—H13	0.9300
N3—C8	1.388 (3)	C14—C15	1.374 (4)
N4—C10	1.330 (3)	C14—H14	0.9300
N4—C16	1.392 (3)	C15—C16	1.388 (3)
N5—C10	1.326 (3)	C15—H15	0.9300
N5—C11	1.392 (3)	C17—O1	1.419 (4)
C1—C2	1.491 (3)	C17—H17A	0.9600
C1—H1A	0.9700	C17—H17B	0.9600
C1—H1B	0.9700	C17—H17C	0.9600
C3—C4	1.375 (3)	Cl1—O3	1.390 (2)
C3—C8	1.382 (3)	Cl1—O4	1.408 (2)
C4—C5	1.375 (4)	Cl1—O5	1.408 (2)
C4—H4	0.9300	Cl1—O2	1.416 (2)
C5—C6	1.379 (4)	Cl2—O7	1.414 (2)
C5—H5	0.9300	Cl2—O9	1.417 (2)
C6—C7	1.377 (4)	Cl2—O6	1.425 (2)
C6—H6	0.9300	Cl2—O8	1.4320 (19)
C7—C8	1.398 (3)	N1—H1	0.86 (3)
C7—H7	0.9300	N2—H2A	0.831 (16)
C9—C10	1.490 (3)	N3—H3A	0.831 (16)
C9—H9A	0.9700	N4—H4A	0.857 (16)
C9—H9B	0.9700	N5—H5A	0.824 (16)
C11—C12	1.382 (3)	O1—H1C	0.805 (17)
			
N1—C1—C2	111.35 (19)	C14—C13—H13	119.2
N1—C1—H1A	109.4	C15—C14—C13	122.4 (2)
C2—C1—H1A	109.4	C15—C14—H14	118.8
N1—C1—H1B	109.4	C13—C14—H14	118.8
C2—C1—H1B	109.4	C14—C15—C16	116.1 (2)
H1A—C1—H1B	108.0	C14—C15—H15	121.9
N3—C2—N2	109.1 (2)	C16—C15—H15	121.9
N3—C2—C1	126.7 (2)	C15—C16—C11	121.5 (2)
N2—C2—C1	124.2 (2)	C15—C16—N4	132.0 (2)
C4—C3—C8	122.6 (2)	C11—C16—N4	106.50 (19)
C4—C3—N2	131.6 (2)	O1—C17—H17A	109.5
C8—C3—N2	105.8 (2)	O1—C17—H17B	109.5
C5—C4—C3	116.3 (2)	H17A—C17—H17B	109.5
C5—C4—H4	121.9	O1—C17—H17C	109.5
C3—C4—H4	121.9	H17A—C17—H17C	109.5
C4—C5—C6	121.6 (3)	H17B—C17—H17C	109.5
C4—C5—H5	119.2	O3—Cl1—O4	110.65 (18)
C6—C5—H5	119.2	O3—Cl1—O5	109.08 (19)
C7—C6—C5	122.8 (3)	O4—Cl1—O5	105.00 (19)
C7—C6—H6	118.6	O3—Cl1—O2	110.09 (15)
C5—C6—H6	118.6	O4—Cl1—O2	112.49 (16)
C6—C7—C8	115.5 (2)	O5—Cl1—O2	109.37 (16)
C6—C7—H7	122.2	O7—Cl2—O9	110.80 (14)
C8—C7—H7	122.2	O7—Cl2—O6	109.72 (15)
C3—C8—N3	106.4 (2)	O9—Cl2—O6	110.25 (16)
C3—C8—C7	121.1 (2)	O7—Cl2—O8	110.00 (14)
N3—C8—C7	132.5 (2)	O9—Cl2—O8	108.70 (14)
N1—C9—C10	110.6 (2)	O6—Cl2—O8	107.30 (13)
N1—C9—H9A	109.5	C1—N1—C9	113.1 (2)
C10—C9—H9A	109.5	C1—N1—H1	104.8 (17)
N1—C9—H9B	109.5	C9—N1—H1	113.8 (17)
C10—C9—H9B	109.5	C2—N2—C3	109.28 (19)
H9A—C9—H9B	108.1	C2—N2—H2A	123.9 (19)
N5—C10—N4	109.1 (2)	C3—N2—H2A	126.5 (19)
N5—C10—C9	125.0 (2)	C2—N3—C8	109.43 (19)
N4—C10—C9	125.9 (2)	C2—N3—H3A	120.5 (18)
C12—C11—N5	132.3 (2)	C8—N3—H3A	129.9 (18)
C12—C11—C16	122.0 (2)	C10—N4—C16	109.0 (2)
N5—C11—C16	105.7 (2)	C10—N4—H4A	123.9 (17)
C13—C12—C11	116.4 (2)	C16—N4—H4A	127.0 (17)
C13—C12—H12	121.8	C10—N5—C11	109.64 (19)
C11—C12—H12	121.8	C10—N5—H5A	121.6 (19)
C12—C13—C14	121.6 (2)	C11—N5—H5A	127.9 (19)
C12—C13—H13	119.2	C17—O1—H1C	115 (2)
			
N1—C1—C2—N3	7.9 (4)	C12—C11—C16—C15	−1.0 (4)
N1—C1—C2—N2	−174.9 (2)	N5—C11—C16—C15	179.7 (2)
C8—C3—C4—C5	0.2 (4)	C12—C11—C16—N4	179.1 (2)
N2—C3—C4—C5	179.7 (2)	N5—C11—C16—N4	−0.1 (2)
C3—C4—C5—C6	−1.5 (4)	C2—C1—N1—C9	−148.2 (2)
C4—C5—C6—C7	2.1 (4)	C10—C9—N1—C1	142.0 (2)
C5—C6—C7—C8	−1.2 (4)	N3—C2—N2—C3	0.7 (3)
C4—C3—C8—N3	−179.8 (2)	C1—C2—N2—C3	−176.9 (2)
N2—C3—C8—N3	0.6 (2)	C4—C3—N2—C2	179.7 (3)
C4—C3—C8—C7	0.7 (4)	C8—C3—N2—C2	−0.8 (3)
N2—C3—C8—C7	−178.9 (2)	N2—C2—N3—C8	−0.3 (3)
C6—C7—C8—C3	−0.2 (4)	C1—C2—N3—C8	177.2 (2)
C6—C7—C8—N3	−179.5 (3)	C3—C8—N3—C2	−0.2 (3)
N1—C9—C10—N5	178.4 (2)	C7—C8—N3—C2	179.2 (2)
N1—C9—C10—N4	−3.2 (4)	N5—C10—N4—C16	0.3 (3)
N5—C11—C12—C13	179.4 (2)	C9—C10—N4—C16	−178.2 (2)
C16—C11—C12—C13	0.3 (4)	C15—C16—N4—C10	−179.9 (2)
C11—C12—C13—C14	1.0 (4)	C11—C16—N4—C10	−0.1 (3)
C12—C13—C14—C15	−1.6 (4)	N4—C10—N5—C11	−0.4 (3)
C13—C14—C15—C16	0.9 (4)	C9—C10—N5—C11	178.1 (2)
C14—C15—C16—C11	0.4 (4)	C12—C11—N5—C10	−178.8 (3)
C14—C15—C16—N4	−179.8 (2)	C16—C11—N5—C10	0.4 (3)

### Hydrogen-bond geometry (Å, °)

**Table:**

*D*—H···*A*	*D*—H	H···*A*	*D*···*A*	*D*—H···*A*
N1—H1···O7^i^	0.86 (3)	2.42 (3)	3.200 (3)	150 (2)
N2—H2A···O8	0.831 (16)	2.15 (2)	2.895 (3)	150 (3)
N2—H2A···O9	0.831 (16)	2.489 (19)	3.233 (3)	150 (2)
N3—H3A···O1	0.831 (16)	1.996 (17)	2.799 (3)	162 (2)
N4—H4A···O1	0.857 (16)	1.985 (17)	2.823 (3)	166 (2)
N5—H5A···O4	0.824 (16)	2.46 (2)	3.197 (4)	150 (2)
N5—H5A···O5	0.824 (16)	2.21 (2)	2.939 (3)	147 (2)
O1—H1C···O6^ii^	0.805 (17)	2.00 (2)	2.765 (3)	159 (3)

Symmetry codes: (i) *x*−1/2, −*y*+1/2, *z*−1/2; (ii) *x*+1/2, −*y*+1/2, *z*−1/2.

### Table 2 Table 2 π–π Stacking interactions (°, Å)

**Table:**

Cg_i_	Cg_j_	Dihedral angle	CCD	Interplanar spacing
Cg1	Cg2^iv^	0.42	3.854 (2)	3.349 (2)
Cg1	Cg4^iv^	0.35	3.557 (2)	3.354 (2)
Cg3	Cg2^iv^	0.85	3.612 (2)	3.360 (2)
Cg3	Cg4^v^	0.36	3.929 (2)	3.453 (2)

CCD is the centroid-to-centroid distance; Cg1 is the centroid of atoms N2/N3/C2/C3/C8; Cg2 is the centroid of atoms N4/N5/C10/C11/C16; Cg3 is the centroid of atoms C3–C8; Cg4 is the centroid of atoms C11–C16. symmetry codes: (iv) 1/2 - x, -1/2 + y,1/2 - z; (v) 1/2 - x, -1/2 + y,1/2 - z.
